# Gall-Bladder Surgery

**Published:** 1892-06

**Authors:** 


### *EDITORIAL.*

---

The office of THE JOURNAL is in rooms 44 and 45 Old Capitol Building.
Address communications relating to the Editorial Department to DR. L. B. GRANDY, Atlanta, Ga., Box 431.
Business communications should be addressed to DR. M. B. HUTCHINS, Atlanta, Ga.
All remittances should be made payable to "ATLANTA MEDICAL AND SURGICAL JOURNAL."
Articles for publication should reach this office not later than the 15th instant.
The editors are not responsible for views expressed by contributors.

---

### GALL-BLADDER SURGERY.

---

This subject has a conspicuous place in the programme of the surgical section at the forthcoming meeting of the American Medical Association in Detroit. The title of the opening address of the Chairman, Dr. J. McFadden Gaston, is "Surgery of the Gall-bladder and Ducts," while there are several other contributions on kindred topics; after the reading of which there is to be a discussion of all the papers by Drs. Vanderveer, Ross, Marcy and Seymour. The session of the section on June 7th will be taken up chiefly with this matter.

The operations upon the gall-bladder and ducts have reached a point, which must claim the favorable consideration of the profession. This new branch of surgery had its origin from an accidental good result in 1867 by Dr. J. S. Dobbs, of Indiana, in cutting into a gall-bladder and discharging the calculi, after which the opening was sutured and dropped into the abdominal cavity. The operation of cholecystotomy has undergone many vicissitudes, but constantly proved successful as a means of temporary relief in biliary obstruction of the cystic duct.

Dr. Carl Langenbuch, of Berlin, initiated the operation of cholecystotomy in 1882, and found benefit in several cases from the entire extirpation of the gall-bladder. Others have also succeeded in thus removing this offending body, and notably Courvoisier, of Basle, and Theriar, of Brussels, have secured good results.

Incision of the common duct for the removal of gall-stones has been done twelve times with only a single death, showing up well for a new and hazardous undertaking.

Two of these operations for removal of calculi from the common duct have been done by American surgeons—Marcy, of Boston, and Vanderveer, of Albany, N. Y., and one by Ross, of Toronto, by incision of the cystic duct. All of these have contributed largely to the literature of gall-bladder surgery; an elaborate paper of the first having been read at the Nashville meeting of the American Medical Association, in May, 1890, while that of the second was presented at the meeting of American Obstetricians and Gynecologists, September, 1891. The paper of the last named has recently appeared in the *American Gynecological Journal*, being an instructive article on "The Dangers of Gall-Stones, with a plea for early laparotomy in obscure abdominal diseases." Ross discusses three procedures: 1, Opening the gall-bladder and fastening it in the wound; 2, opening the gall-bladder and dropping it back into the abdomen; 3, operation on the common duct itself, and supplemental proceedings to overcome the obstruction when certain conditions are present. He states that impaction of stones in the ducts—cystic, hepatic or common—is a formidable complication. If no stones be found in the gall-bladder, the common duct should always be explored. He says further that incision directly into the duct, if it can be carried out, and if the necessary sutures can be subsequently applied, is certainly the best procedure, if the stone cannot be crushed.

A most important field of investigation has been entered upon more earnestly by surgeons latterly in the development of the possibilities of anastomosis of the gall-bladder and the common duct with the intestinal canal. Whatever views may be held in regard to the functions of bile in the process of digestion and assimilation, it is known to subserve an important purpose in promoting the normal state of the contents of the small and large intestines. It is, therefore, considered important to restore the bile to the alimentary canal by the process of cholecystenterostomy when the common duct becomes obstructed.

Courvoisier, of Basle, has published a very complete history of all the various steps taken by different surgeons throughout the world for the relief of biliary obstruction, and some one would confer a good service to the profession by translating the German text into English. Among other matters he treats more fully of effecting a communication from the gall-bladder into the duodenum and the intestinal canal, than has been done by any previous writer. The experiments of Gaston* for the illustration of this operation are fully described; and the record of the different operations on the human subject, which have been successfully done, encourages the expectation that the day is not far distant when duodeno-cholecystostomy and cholecystenterostomy must be generally adopted for providing an artificial opening into the intestines, when the common bile duct is permanently occluded.

The ordinany operation of cholecystotomy gives an outlet externally to the bile, by which it is lost, whereas the new operation of restoring it into the original channel, turns it to account in promoting the functions of the intestinal canal, and is one of the most important advances made in surgery.

---

* First published in this JOURNAL September and October, 1884, with the title "Experimental Cholecysto-Cystotomy," and September and November, 1885, with the title "Experimental Duodeno-Cholecystostomy."

